# The use of implementation science frameworks to promote maternal and child health delivery programs in Nigeria

**DOI:** 10.3389/frhs.2026.1804247

**Published:** 2026-06-04

**Authors:** Uju Maryanne Onuorah, Moses Ifeatu Nwuzoh, Francisca Ogochukwu Onukansi, Collins Chibueze Anokwuru, Ogechi Vinaprisca Ikhuoria, Chidera Gabriel Obi, Stanley Chinedu Eneh

**Affiliations:** 1 Liverpool School of Tropical Medicine, Liverpool, United Kingdom; 2 Department of Pharmacy and Health and Nutrition Sciences, University of Calabria, Calabria, Italy; 3 Department of Nutrition and Dietetics, University of Nigeria, Nsukka/Enugu, Nigeria; 4 Youth in Research Hub, Enugu, Enugu State, Nigeria; 5 Department of Public Health, Sheffield Hallam University, Sheffield, United Kingdom; 6 Department of Public Health, Federal University of Technology Owerri, Owerri, Nigeria; 7 Department of Behavioral and Community Health, University of Maryland School of Public Health, College Park, MD, United States; 8 Department of Parasitology and Entomology, Nnamdi Azikiwe University, Awka, Anambra, Nigeria; 9 Interdisciplinary Health Sciences, The University of Texas at El Paso, El Paso, TX, United States

**Keywords:** CFIR, diffusion of innovations, health policy, implementation science, maternal and child health, Nigeria, RE-AIM

## Abstract

Maternal and Child Health (MCH) remains a significant public health concern in Nigeria, with high maternal and child mortality rates. Although effective, evidence-based interventions exist, their impact is often constrained by implementation gaps driven by weak health systems, limited resources, and sociocultural factors. This perspective article highlights the untapped potential of implementation science (IS) frameworks to improve the planning, implementation, and evaluation of MCH programs in Nigeria. This study examines four key implementation science frameworks: the Consolidated Framework for Implementation Research (CFIR), RE-AIM, Promoting Action on Research Implementation in Health Services (PARiHS), and the Diffusion of Innovations and explores their relevance to Nigeria's MCH system using case examples. Key challenges include limited IS capacity, weak health system structures, and sociocultural barriers. However, opportunities exist in digital health innovations, workforce development, and multi-sectoral collaboration. Integrating implementation science into national MCH strategies could enhance their effectiveness and accelerate progress toward achieving Sustainable Development Goal 3.

## Introduction

Maternal and child health (MCH) remains a significant public health challenge in Nigeria, where maternal and neonatal mortality rates are among the highest globally. The World Health Organization (WHO) estimates the country's maternal mortality ratio at 512 per 100,000 live births, accounting for approximately 20% of global maternal deaths (1). Similarly, the infant mortality rate remains high at 67 deaths per 1,000 live births, driven by limited access to quality healthcare, low utilization of antenatal services, and preventable conditions (2). In response, several national policies have been introduced, including the 2014 National Health Act and the Integrated Maternal, Newborn, and Child Health (IMNCH) Strategy, aimed at strengthening service delivery and coordinating interventions. Despite these efforts, persistent structural, systemic, and sociocultural challenges continue to limit the effectiveness and scalability of MCH programs (2, 3).

Implementation science (IS) offers a structured approach to addressing these gaps by focusing on how evidence-based interventions can be effectively translated into real-world practice. However, in many low- and middle-income country (LMIC) settings, including Nigeria, the application of IS frameworks remains fragmented and insufficiently adapted to local health system realities. This article argues that no single implementation science framework is sufficient to address the complexity of maternal and child health systems in Nigeria. Instead, an integrated and context-sensitive approach is required, one that combines the strengths of multiple frameworks to improve program design, implementation, evaluation, and community uptake. This study focuses on the CFIR, RE-AIM, PARiHS, and Diffusion of Innovations frameworks and examines how these can be applied together in the Nigerian MCH context using case examples to inform policy and practice.

## Brief overview of implementation science frameworks

Implementation Science frameworks provide a systematic way of embedding evidence-based practice (EBP) in real-world healthcare settings. These theories and models offer a way to identify potential barriers, facilitators, and central determinants of the success of public health interventions and programs (4, 5). IS systems provide mechanisms for the systemic implementation and maintenance of MCH interventions, especially in the complicated healthcare system in Nigeria (6). These frameworks can be classified as process models, determinant frameworks, classic theories, or evaluation frameworks (7). Data-guided process models such as the Knowledge-to-Action (KTA) framework guide the flow of research into action, policy, and practice (8). Determinant frameworks, including the Consolidated Framework for Implementation Research (CFIR), provide a view of the structural framework that may support or obstruct the implementation of a program (9).

## Relevant IS frameworks for maternal and child health programs in Nigeria

Several implementation science (IS) frameworks have been developed to support the translation of evidence-based interventions into practice (4, 7). However, no single framework fully captures the complexity of health system challenges, particularly in low- and middle-income countries such as Nigeria (13). The CFIR focuses on identifying contextual determinants of implementation across multiple levels of the health system (9). The PARiHS emphasizes the interaction between evidence, context, and facilitation during the implementation process (11). The RE-AIM provides a structured approach for evaluating program reach, effectiveness, adoption, implementation, and sustainability (10). The Diffusion of Innovations framework explains how new practices are adopted within communities and social systems (12).

While several implementation science frameworks exist, this article focuses on the CFIR, RE-AIM, PARiHS, and Diffusion of Innovations frameworks because they capture key and complementary domains of implementation relevant to maternal and child health systems. These include contextual analysis, implementation processes, evaluation of outcomes, and community-level adoption. Other implementation science frameworks, such as the COM-B, Proctor's implementation outcomes framework, and the EPIS, also offer valuable insights but were not included in the main analysis to maintain conceptual clarity and focus. Instead, this study prioritizes frameworks that together provide a comprehensive and practical approach to understanding and improving implementation across multiple levels of the Nigerian health system.

These frameworks offer a multi-level and integrated approach that is particularly relevant to Nigeria's maternal and child health (MCH) system, where implementation challenges occur across policy, health system, and community levels (13). Rather than functioning as isolated tools, they can be applied sequentially and complementarily. For example, the CFIR can be used at the initial stage to assess contextual barriers, such as facility readiness, workforce capacity, and governance constraints (9). The PARiHS can then guide the implementation process by ensuring alignment between available evidence, the local context, and facilitation strategies (11). The RE-AIM can be applied to evaluate program performance across key dimensions, including reach and sustainability (10), while the Diffusion of Innovations framework can support community-level adoption by addressing sociocultural factors and leveraging local influencers (12).

This integrated approach enables policymakers and implementers to move beyond fragmented strategies toward a more coordinated approach that aligns diagnosis, implementation, evaluation, and community engagement. Such an approach is critical for improving the effectiveness, scalability, and sustainability of MCH interventions in Nigeria. Table 1 presents a comparative summary of these frameworks, highlighting their key domains, strengths, and relevance to the Nigerian context and illustrating how they can be strategically applied to strengthen MCH program delivery (9–12).

**Table 1:** A comparison of four key implementation science frameworks and their application in the context of Nigeria's maternal and child health (MCH).

Framework	Key domains	Strengths	Implementation in the context of MCH in Nigeria
CFIR (Consolidated Framework for Implementation Research)	Intervention characteristics, Outer setting, Inner setting, Characteristics of individuals, Process (e.g., multi-level contextual diagnosis)	Strong diagnosis of the implementation context; identifies barriers and enablers.	Helps assess health system barriers, enablers, and guides the adaptation of MCH programs to local contexts (e.g., MSS)
RE-AIM	Reach, Effectiveness, Adoption, Implementation, Maintenance (e.g., scalability and evaluation)	Useful for strong planning, evaluating, and scaling of real-world impacts.	Evaluates the scalability and sustainability of MCH programs in rural and urban settings
PARiHS (Promoting Action on Research Implementation in Health Services)	Evidence, Context, Facilitation (e.g., evidence translation with facilitation)	Supports and facilitates evidence translation into real-world settings.	Supports the implementation of midwifery-led care models and evidence-based antenatal care
Diffusion of Innovations	Innovation, Communication channels, Time, Social system (e.g., Behavioral and cultural adoption pathways).	Emphasizes how innovations are adopted across communities; identifies opinions, social behaviors, leaders, and influences.	Suitable for promoting the uptake of maternal health behaviors through community engagement and peer influence

Adapted from Damschroder et al., Glasgow et al., Kitson et al., Rogers (9–11).

**Figure 1:**
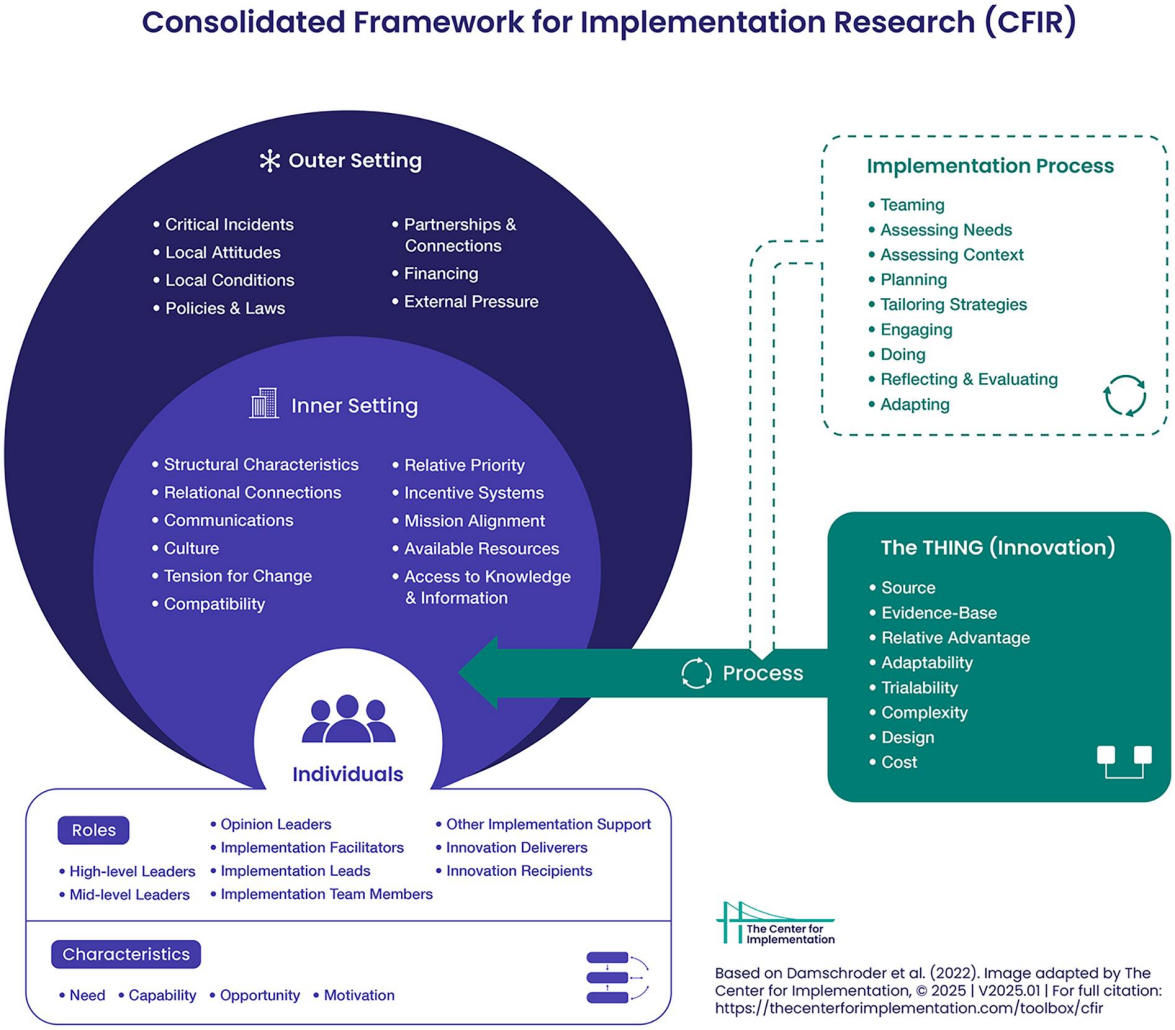
Domains of the consolidated framework for implementation research (CFIR). Adapted from Damschroder et al. (9).

**Figure 2:**
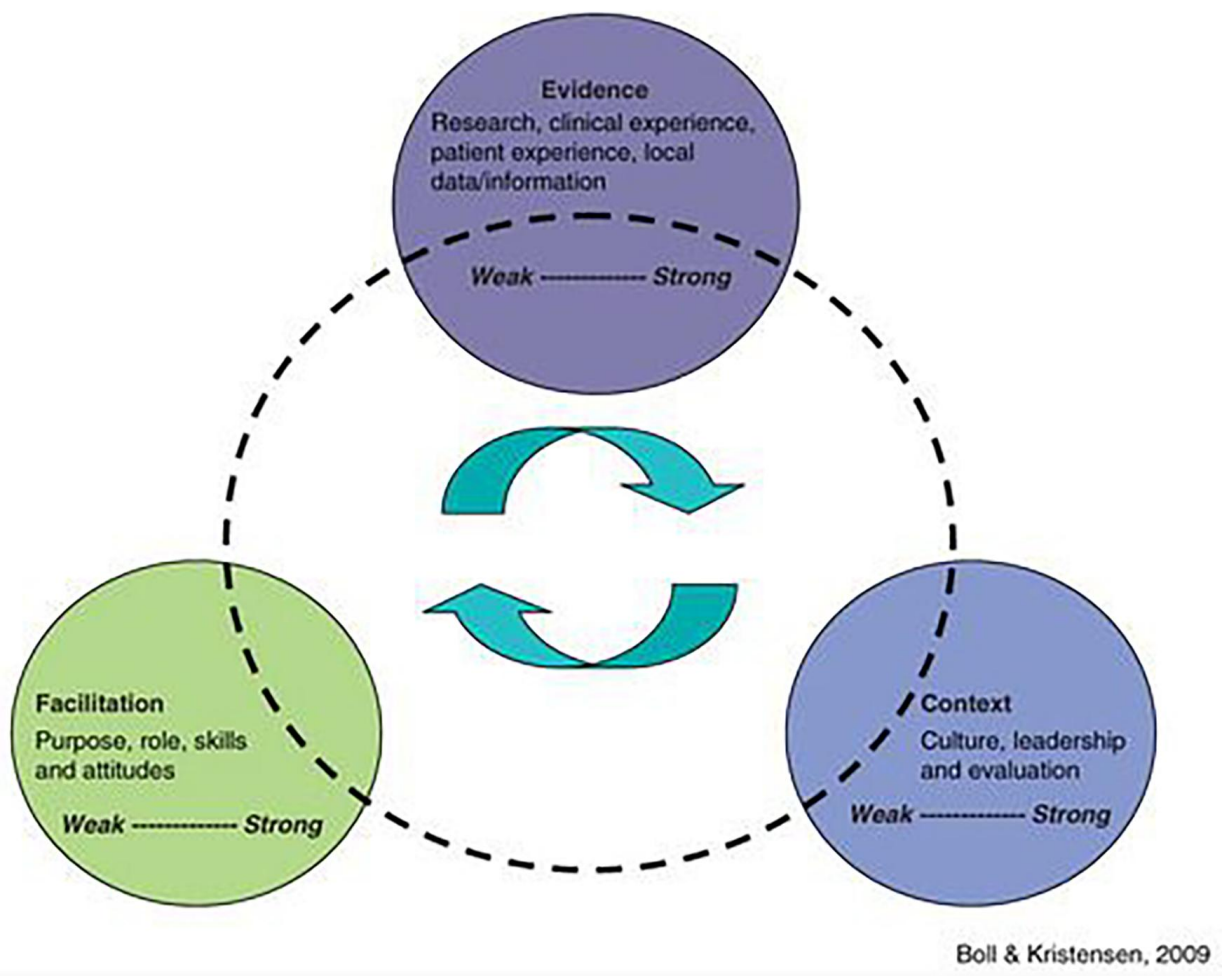
The PARIHS (promoting action on research implementation in health services) framework demonstrating the relationship between evidence, context, and facilitation in implementation outcomes. Adapted from Kitson et al. (11).

**Figure 3:**
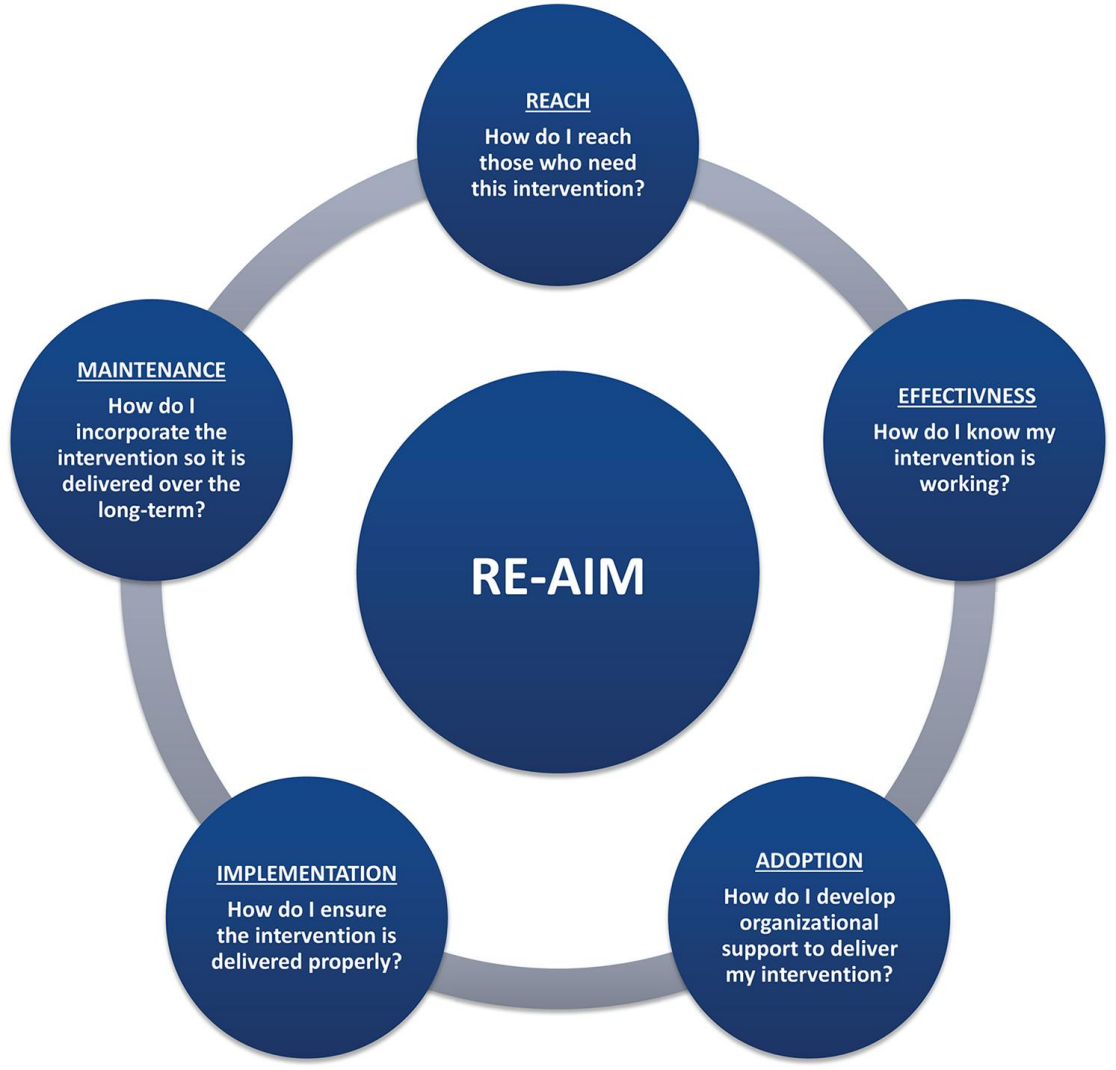
The RE-AIM framework, highlighting the key domains of reach, effectiveness, adoption, implementation, and maintenance. Adapted from Glasgow et al. (10).

The RE-AIM framework, as shown in Figure 1, evaluates interventions based on reach, effectiveness, adoption, implementation, and maintenance and is particularly useful for assessing scalability and long-term impact (10). Similarly, the CFIR framework, shown in Figure 2, provides a structured approach to examining contextual factors that influence implementation at various levels of the health system (9). The PARiHS framework, shown in Figure 3, emphasizes the dynamic interaction between evidence, context, and facilitation, highlighting the importance of supportive implementation processes (11). In contrast, the Diffusion of Innovations framework focuses on how new practices spread within communities, making it especially relevant for addressing sociocultural barriers to the uptake of maternal health services.

While these frameworks offer valuable guidance for strengthening MCH program implementation, their application in low-resource settings such as Nigeria is not without limitations. For example, the CFIR framework, although comprehensive in diagnosing contextual barriers, can be methodologically complex and resource-intensive, making it difficult to apply routinely in under-resourced health systems (9). Similarly, the RE-AIM provides a robust structure for evaluating implementation outcomes, but its effectiveness depends on the availability of reliable data systems, which are often weak in many Nigerian health facilities (10, 13). The PARiHS framework emphasizes facilitation and organizational context, but its successful application requires skilled personnel and strong institutional support, both of which may be limited in practice (11). In addition, while the Diffusion of Innovations framework offers valuable insights into community-level adoption, it may not fully account for structural constraints such as poverty, access barriers, and health system inefficiencies (12, 21). These limitations highlight the need for adapting implementation science frameworks to local realities and reinforce the importance of using them in a complementary and flexible manner rather than as standalone tools.

In practice, the choice between using a single framework or combining multiple frameworks depends on the complexity of the implementation context and the goals of the intervention (4, 7). A single framework may be sufficient when addressing a specific aspect of implementation, such as identifying contextual barriers (e.g., the CFIR) or evaluating program outcomes (e.g., the RE-AIM) (9, 10). However, in more complex health system settings, such as in Nigeria, where challenges span policy, health systems, and the community level, combining frameworks may provide a more comprehensive approach (13). For example, the CFIR can be used to assess contextual barriers, the PARiHS can guide implementation processes, the RE-AIM can evaluate outcomes, and the Diffusion of Innovations framework can support community uptake (9–12). While combining frameworks offers broader insights, it may also increase complexity and require greater capacity and coordination, highlighting the need for a context-sensitive and pragmatic application.

## Key implementation challenges in Nigeria

The application of implementation science (IS) frameworks in maternal and child health (MCH) programs in Nigeria is constrained by several systemic, structural, and sociocultural challenges. A major barrier is the limited awareness of and capacity among healthcare providers and policymakers to apply IS frameworks in routine practice. Many professionals are unfamiliar with tools such as CFIR and RE-AIM, resulting in the weak integration of research evidence into program implementation (13). Evidence from the SURE-P/MCH realist evaluation further highlights gaps in training and systems support, which limited the ability of frontline workers to effectively implement and sustain interventions (14). Resource and infrastructure limitations also pose significant challenges, including shortages of skilled personnel, inconsistent funding, and weak supply chain systems. For example, the application of the CFIR framework to a quality improvement initiative in Kano State identified key operational barriers such as inadequate cold chain systems, frequent staff rotation, and irregular supervision, all of which hindered implementation effectiveness (18).

In addition, sociocultural factors continue to influence the uptake of MCH interventions. Traditional beliefs, gender norms, and low levels of health literacy often limit the utilization of facility-based maternal health services. Studies of the Midwives Service Scheme (MSS) have shown that reliance on home births and cultural practices reduces the uptake of skilled birth attendance in some communities (15). Governance and policy-related challenges further complicate implementation efforts, as fragmentation across federal, state, and local levels of the health system often leads to poor coordination and inconsistent program delivery. For instance, the SURE-P/MCH program experienced disruptions due to policy discontinuity and funding withdrawal, which undermined implementation fidelity and sustainability (14). Data limitations also remain a critical constraint, with weak health information systems and poorly defined indicators hindering effective monitoring and evaluation. In the Lagos CQI initiative on antenatal anemia screening, limited baseline data initially constrained planning and decision-making, highlighting the importance of strengthening data systems for effective implementation (16).

## Case examples: application of IS frameworks in Nigeria’s MCH programs

The application of implementation science (IS) frameworks in Nigeria demonstrates their potential to address persistent challenges in maternal and child health (MCH) program delivery. Their value lies not only in their use but also in how they diagnose implementation barriers, guide context-specific strategies, and generate actionable lessons. One of the most illustrative examples is the Subsidy Reinvestment and Empowerment Programme for Maternal and Child Health (SURE-P/MCH), which applied a realist evaluation approach to examine how and why interventions worked in specific contexts (14). The program combined supply-side strategies such as deployment of skilled health workers and facility upgrades with demand-side interventions, including conditional cash transfers. The evaluation identified key barriers, including weak facility readiness and variability in community engagement, and demonstrated that program effectiveness depended heavily on contextual factors, such as political commitment, staffing levels, and community participation. This highlights the importance of context-sensitive implementation strategies for sustaining MCH interventions.

Similarly, the application of the Consolidated Framework for Implementation Research (CFIR) to a quality improvement initiative targeting missed opportunities for vaccination in Kano State illustrates how structured frameworks can guide implementation (18). The CFIR was used to identify barriers, such as poor workflow processes, inadequate training of health workers, and weak community mobilization, which informed the use of Plan–Do–Study–Act cycles to improve service delivery practices. In Lagos State, a Continuous Quality Improvement approach using the Diagnose–Intervene–Verify–Adjust model was applied to address gaps in antenatal anemia screening (16). This intervention emphasized participatory implementation, enabling local health workers to identify bottlenecks, such as delays in laboratory turnaround time and weak referral systems, and to co-develop adaptive solutions that improved screening uptake and service coordination.

At a broader systems level, the Nigeria Implementation Science Alliance (NISA) applied the Exploration, Preparation, Implementation, and Sustainment (EPIS) framework to establish Model Innovation and Research Centers across multiple regions (17). This initiative addressed challenges related to weak research infrastructure and limited coordination by strengthening stakeholder engagement, building institutional capacity, and integrating research into practice. In addition, recent evaluations of Nigeria's adoption of the WHO antenatal care model used implementation outcome frameworks to assess program fidelity across multiple states (19, 20). These studies identified key barriers, such as inconsistent supervision and supply chain disruptions, while demonstrating that higher implementation fidelity is associated with improved maternal outcomes. Collectively, these examples show that implementation science frameworks can be effectively applied across diverse levels of Nigeria's health system to improve program design, delivery, and sustainability.

## Strategic opportunities and policy implications

Despite these challenges, implementation science frameworks present important opportunities to strengthen maternal and child health (MCH) programs in Nigeria. One key opportunity lies in capacity building. Experiences from programs such as SURE-P/MCH have demonstrated that even limited exposure to implementation approaches can enable local teams to begin applying theory-driven strategies and adapting interventions to their context, highlighting the potential for scaling IS training within the health system (14). Evidence from other low-resource settings has also shown that targeted implementation science training can strengthen the research and practice abilities of health professionals (22). Implementation of science frameworks has also provided tools for identifying and addressing system bottlenecks early on. In Kano State, for example, the use of the CFIR framework enabled implementers to detect operational weaknesses and design adaptive quality improvement strategies, demonstrating how structured approaches can improve program performance even in resource-constrained settings (18).

Community engagement represents another critical opportunity. The application of the Diffusion of Innovations framework principles has shown that involving local influencers, traditional birth attendants, and community leaders can significantly improve the acceptance and uptake of maternal health services. Evidence from conditional cash transfer programs linked to SURE-P has indicated that culturally aligned strategies can enhance service utilization and program sustainability (14). Sociocultural factors influencing health-seeking behavior in Nigeria have further highlighted the importance of context-sensitive approaches to program implementation (23). Furthermore, implementation science can support resilience within health systems by enabling adaptation to policy and funding changes. The experience of the Nigeria Implementation Science Alliance (NISA) has illustrated how national coordination, stakeholder engagement, and the use of structured implementation strategies can sustain research and program activities despite resource and governance constraints (17).

Finally, IS frameworks offer an opportunity to strengthen data-driven decision-making and innovation. Participatory approaches used in CQI initiatives in Lagos have demonstrated that, even in low-data environments, local teams can develop context-specific indicators and monitoring systems, fostering continuous learning and improvement (16). In addition, digital health innovations have shown the potential to improve access to maternal and child health services in sub-Saharan Africa, particularly in resource-constrained settings (24). Implementation research approaches can further support the systematic integration of evidence into policy and practice across health systems (25). These opportunities highlight the potential of embedding implementation science in policy and practice to improve the effectiveness, scalability, and sustainability of MCH interventions in Nigeria.

## Recommendations and conclusion

To strengthen maternal and child health (MCH) program delivery in Nigeria, it is necessary to deliberately integrate implementation science (IS) frameworks into national health policies and program design. This should be led by key institutions such as the Nigerian Federal Ministry of Health (FMoH), State Ministries of Health, and the National Primary Health Care Development Agency (NPHCDA), with support from academic institutions and development partners. This includes investing in workforce capacity to improve familiarity with IS methods, strengthening health information systems to support data-driven decision-making, and promoting the use of context-sensitive implementation strategies across various levels of the health system. In addition, greater emphasis should be placed on community engagement approaches that leverage local structures and sociocultural dynamics to improve the uptake and sustainability of MCH interventions. Multi-sectoral collaboration involving the government, academia, and development partners will also be essential for supporting coordinated and scalable implementation efforts.

Implementation science offers a critical pathway for strengthening maternal and child health (MCH) programs in Nigeria by addressing persistent gaps between evidence and practice. However, the complexity of the Nigerian health system necessitates moving beyond the isolated use of individual frameworks toward a more integrated and context-sensitive approach. As demonstrated in this article, frameworks such as the CFIR, PARiHS, RE-AIM, and Diffusion of Innovations provide complementary strengths that can be applied to program design, implementation, evaluation, and community engagement. Evidence from Nigerian case studies further shows that, when applied in adaptive and context-aware ways, these frameworks can enhance program effectiveness, scalability, and sustainability, although their impact remains constrained by limitations in capacity, data systems, governance, and sociocultural alignment. Embedding implementation science within national health strategies, therefore, provides a practical and scalable pathway to improve the delivery and long-term impact of MCH interventions in Nigeria and similar low-resource settings.

## Data Availability

The original contributions presented in the study are included in the article/Supplementary Material, further inquiries can be directed to the corresponding author.
